# Editorial: Fast transit, crew health, and performance on extended duration space missions

**DOI:** 10.3389/fpubh.2023.1318619

**Published:** 2024-02-20

**Authors:** Annette Louise Sobel

**Affiliations:** University of Missouri, Columbia, MO, United States

**Keywords:** crew health, crew safety, radiation mitigation, extended duration space mission, nuclear rocket

NASA is currently planning a human flight to Mars, which will require approximately a forty-million-mile journey, which is over 150 times longer than the distance between the Earth and Moon. This journey will involve prolonged transit outside of the Earth's magnetosphere, where intense radiation levels threaten the crew's performance, health, and survival. Advanced forms of nuclear rocketry are under development now to transit this distance as rapidly as feasible, and thereby minimize the total radiation dose to the crew.

The protection of the crew's health and performance requires a fully interdisciplinary scientific and engineering approach. New biomedical developments may make the crews less susceptible to space radiation, and other environmental threats. Advanced crew monitoring and shielding may provide additional protection strategies and countermeasures. Current nuclear thermal rockets can minimize this Earth–Mars transit time to roughly 1 month in each direction, and future fission-fragment nuclear rocket propulsion may reduce this time to < 1 week, but this will require new accomplishments in advanced materials and nuclear technology. It is essential that these missions be conducted during periods of minimal particle radiation from the Sun. Space weather and solar flare activity may be currently forecast out to 45 days from the present. We seek to explore and identify biotechnology that protects human health, new shielding, monitoring, and counter-measure technologies, new methods that refine and extend space weather forecasting, and especially new propulsion technologies that greatly reduce the transit time to Mars and beyond.

Original research articles published in this Research Topic publication begin to address some of the most complex interdisciplinary challenges to preserving crew health on long missions to be conducted outside of the Earth's magnetosphere, such as from the Earth to Mars. Topics include adaptive, mission-centric medical standards, metagenomic anomaly detection, genomic age-associated risks to crew health, and application of advanced analytics to ensure crew health maintenance. The most effective strategy for long-duration human crew missions is to go exceptionally fast, to minimize the crew's exposure to the radiation environment. Currently NASA and DOE are carefully considering two different types of nuclear rocketry for such missions, one that utilizes thermal expansion of a liquid hydrogen accelerant, and the other that utilizes thermionic conversion of the reactor's heat flux directly to electricity that is subsequently used for ion rocket acceleration. In this Research Topic, we introduce a much more advanced manifestation of a fission fragment rocket that can only be used outside of the Earth's atmosphere, but that provides far greater performance and assurance of reduced radiation exposure than the nuclear rockets that are currently under consideration for the human mission to Mars.

## Author contributions

AS: Writing—original draft, Writing—review & editing.

